# Unveiling the Mystery of the Stimulatory Effects of Arecoline: Its Relevance to the Regulation of Neurotransmitters and the Microecosystem in Multi-Ecological Intestinal Sites

**DOI:** 10.3390/ijms26073150

**Published:** 2025-03-28

**Authors:** Junxi Shen, Mengsi Zhou, Nenqun Xiao, Zhoujin Tan, Xuejuan Liang

**Affiliations:** 1 School of Traditional Chinese Medicine, Hunan University of Chinese Medicine, Changsha 410208, China; shenjunxi816@163.com (J.S.); mengsiz26@163.com (M.Z.); 2 Hunan Key Laboratory of Traditional Chinese Medicine Prescription and Syndromes Translational Medicine, Hunan University of Chinese Medicine, Changsha 410208, China; 003793@hnucm.edu.cn; 3 Institute of Innovative Traditional Chinese Medications, Hunan Academy of Chinese Medicine, Changsha 410013, China

**Keywords:** *Areca catechu* L., betel nut, arecoline, neurotransmitter, intestinal microbiota, short-chain fatty acids, microecosystem

## Abstract

The dried ripe seeds and pericarp of *Areca catechu* L., a palm species, possess significant economic value. Masticating betel nut is also a long-standing and widely prevalent lifestyle habit rooted in history, known for its stimulating effect. This effect stems primarily from arecoline, the principal active compound in betel nut. This study investigates the potential mechanisms underlying the stimulating effects of arecoline, focusing on neurotransmitters, neurotrophic factors, and the microecosystem in multi-ecological intestinal sites. After arecoline intervention in mice, significant changes were observed in locomotor activity. The levels of dopamine (DA) in liver tissue and 5-hydroxytryptamine (5-HT) in brain tissue were significantly reduced. There was a significant increase in microbial activity in the feces and in the level of n-valeric acid in the intestinal content. At the genus level, the relative abundance of *Clostridium* was significantly reduced, whereas the relative abundances of *Helicobacter* and *Aquincola* were markedly increased. *Helicobacter*, *Aquincola*, *Faecalibaculum*, and *Liquorilactobacillus* were signature genera in the arecoline-treated group. The 5-HT level was significantly negatively correlated with the abundance of the signature genera *Aquincola*, *Helicobacter*, and *Liquorilactobacillus* in the arecoline group. The ingestion of arecoline can alter the behavioral patterns of mice, causing significant changes in the 5-HT levels in brain tissue and exerting regulatory effects on the microecosystem in multi-ecological intestinal sites. These findings will provide a reference for the future development and utilization of betel nut.

## 1. Introduction

Betel nut, the fruit of *Areca catechu* L., a tropical plant from the palm family, is primarily found in tropical regions of Asia and the Americas. Globally, it ranks as the fourth most prevalent psychoactive substance consumed, trailing only behind tobacco, alcohol, and caffeine [1]. Masticating betel nut elicits sensations of bliss, exhilaration, physical warmth, heightened vigilance, and augmented work efficiency [2]. Approximately 600 million individuals worldwide indulge in the consumption of betel nut, particularly in East and South Asia, East Africa, and certain areas of the tropical Pacific [3]. Betel nut exhibits diverse pharmacological activities: in the digestive system, it promotes digestion and eliminates gastrointestinal parasites; in the nervous system, it exhibits central nervous system stimulant and anticonvulsant properties; and in the cardiovascular system, it possesses blood pressure-regulating activity. Additionally, it is used in the treatment of diabetes, stomatitis, and urinary system disorders [4]. Betel nut presents a diverse array of bioactive constituents, encompassing alkaloids, polyphenolic compounds, polysaccharides, amino acids, fatty acids, and tannins [5]. Among these, arecoline is the primary active component and is a naturally occurring psychoactive alkaloid. Arecoline can mimic various effects of acetylcholine, such as muscarinic actions that stimulate glandular secretion, nicotine-like effects that reduce pressure in the intestine and blood vessels, and effects on ganglia and striated muscles, known as “nicotinic effects” [6]. As a partial agonist of nicotinic and muscarinic acetylcholine receptors, it acts as a stimulant for both the central and the autonomic nervous systems, leading to increased subjective feelings of well-being, alertness, and endurance [7]. In previous studies, our team investigated the stimulating effects and acute toxicity of arecoline [8]. We found that arecoline significantly delayed the onset of sleep in mice. Moreover, as the concentration of arecoline increased, its anti-hypnotic effect became more pronounced. The lethal dose 50 (LD50) of arecoline was determined to be 430 mg/kg, with a 95% confidence interval of 295–626 mg/kg.

Recently, an expanding array of studies has illuminated the intimate link between intestinal microbiota and central nervous system functionalities. Dopamine (DA), a pivotal excitatory neurotransmitter, holds a significant position within the reward pathway, influencing motivation, pleasure sensations, and reward-based learning [9]. The intestinal microbiota promotes the synthesis of neurotransmitters and their precursors by producing enzymes and communicates with enteroendocrine cells through its metabolites, thereby regulating the production and release of neurotransmitters [10]. Specific intestinal microorganisms have the capability to produce 3,4-Dihydroxyphenyl L-Alanine (L-DOPA), which traverses into the central nervous system via the bloodstream and is converted into DA. This conversion process is vital for sustaining adequate DA levels [11,12]. Additionally, the intestinal microbiota regulates vitamin B production, influencing autism-like behaviors in EphB6-deficient mice, with a key mechanism involving the regulation of DA levels [13]. The intestinal microbiome contributes to the production of fatty acid amide metabolites in the intestine, stimulating transient receptor potential vanilloid 1+ (TRPV1+) sensory neurons that express the endocannabinoid receptor cannabinoid receptor-1 (CB1), thereby sending motor-induced afferent signals to the brain and promoting the downregulation of striatal monoamine oxidase (MAO) expression. This downregulation of MAO helps to elevate the DA levels, subsequently enhancing the motor ability [14]. 5-Hydroxytryptamine (5-HT), more commonly referred to as serotonin, is a crucial neurotransmitter and biogenic amine that primarily exerts its effects within the central nervous system and gastrointestinal tract [15]. The majority of the body’s 5-HT is found in the gastrointestinal tract, where the intestinal microbiota plays a pivotal role in regulating the host’s 5-HT levels. Native spore-forming bacteria, present in both mouse and human intestine, enhance 5-HT biosynthesis in colonic enterochromaffin cells [16]. *Roseburia intestinalis* increases the 5-HT levels in the brain and colon by promoting the expression of tryptophan hydroxylase-2 or -1, significantly improving the depression-like behavior in mice induced by chronic restraint stress [17]. Additionally, members of the Enterobacteriaceae and Enterobacteriales families can ferment fiber to produce short-chain fatty acids (SCFAs) such as acetic acid and formic acid, which in turn stimulate 5-HT biosynthesis in enteric chromaffin cells. Elevated 5-HT levels in this context may be linked to an increased risk of schizophrenia [18]. In addition to DA and 5-HT, the intestinal microbiota can directly or indirectly produce various neurotransmitters such as γ-aminobutyric acid (GABA), acetylcholine, and norepinephrine [19]. GABA stands out as a pivotal inhibitory neurotransmitter, functioning by modulating neuronal excitability through the engagement of GABA receptors, thereby exerting suppressive effects on neuronal activity [20]. The intake of *Lactobacillus rhamnosus* has been shown to modulate emotional behavior and central GABA receptor expression in mice via the vagus nerve [21]. Brain-derived neurotrophic factor (BDNF) is a neurotrophic factor belonging to the neurotrophin family [22]. These neuromodulators are indispensable for maintaining mood stability, attention levels, and alertness.

To understand the stimulating mechanism of arecoline, it is crucial to consider the role of the intestinal microecosystem, particularly how it mediates neuroactive effects by influencing neurotransmitter levels. However, the specific mechanism of arecoline’s action in the central nervous system and the involvement of the intestinal microbiota in this mechanism remain incompletely elucidated. Hence, this research advances the hypothesis that arecoline potentially modulates central nervous system functionality by altering the levels of neurotransmitters or neurotrophic factors, with concurrent shifts in the intestinal microecosystem being intricately tied to arecoline’s neural mechanisms. Changes in the structure and function of the intestinal microbiota induced by arecoline—such as shifts in microbial composition or metabolite production—may, in turn, influence the balance of neurotransmitters, thereby forming a feedback loop between the gut and the central nervous system. Elucidating this mechanism not only enhances our understanding of arecoline’s stimulating effects but also may pave the way for novel therapeutic strategies for related neurodegenerative diseases.

## 2. Results

### 2.1. Effects of the Arecoline Solution on Mental State and Spontaneous Activity in Mice

The mental state and spontaneous activity of the mice were observed using the open-field test, as shown in Table 1. The test was conducted on days 7, 14, 21, and 28 of the experiment. The results showed no significant differences between the two groups of mice in total distance traveled, average speed, time spent in fast movement, and number of center entries on days 7 and 14. On day 21, it was found that the mice in the arecoline group exhibited significantly prolonged fast movement time and an increased number of center entries (*p* < 0.05). On day 28, the number of center entries was significantly higher for the arecoline group compared to the blank control group (*p* < 0.05).

### 2.2. Effects of the Arecoline Solution on the GABA, DA, 5-HT, and BDNF Levels in the Liver and Brain Tissues of Mice

**Figure 1:**
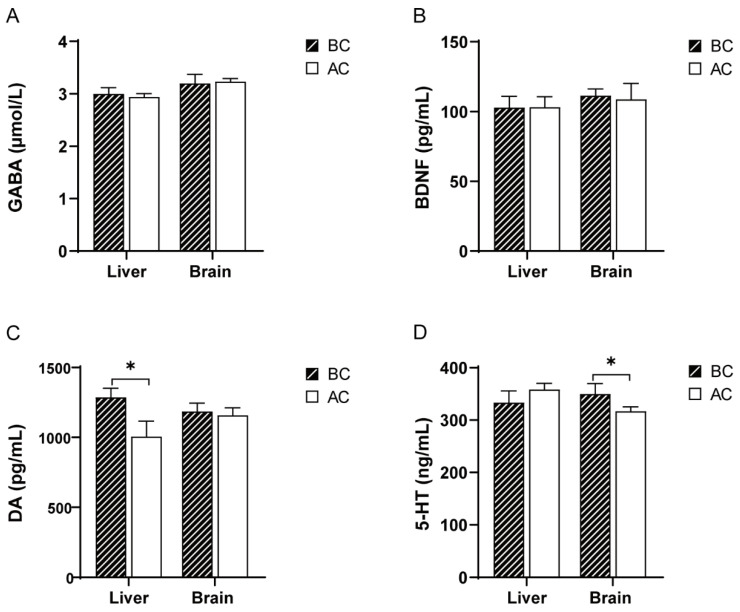
Effects of arecoline solution on GABA, BDNF, DA, and 5-HT in mice. (**A**) GABA, (**B**) BDNF, (**C**) DA, (**D**) 5-HT. * *p* < 0.05. BC: blank control group; AC: arecoline group.

Compared to the blank control group, there were no significant differences in the GABA and BDNF levels in the liver and brain tissues of the arecoline group (*p* > 0.05); the DA levels were significantly increased in the liver tissue (*p* < 0.05), but there was no significant difference in the DA levels in the brain tissue (*p* > 0.05); the 5-HT levels were significantly decreased in the brain tissue (*p* < 0.05), while there was no significant difference in the 5-HT levels in the liver tissue (*p* > 0.05) (Figure 1).

### 2.3. Effects of the Arecoline Solution on the Morphology of the Liver and Brain Tissues in Mice

**Figure 2:**
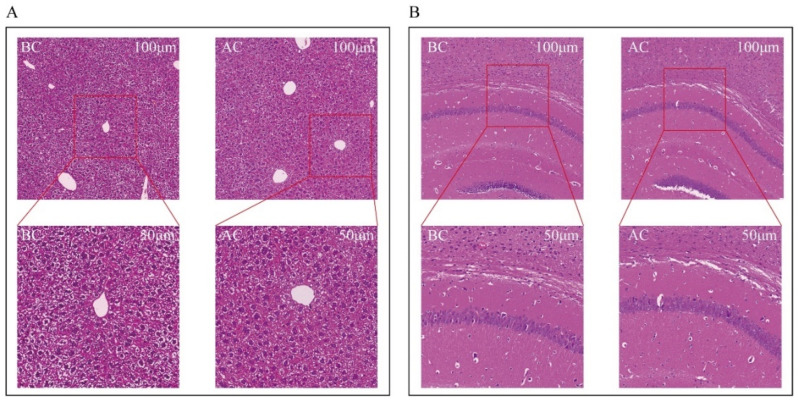
Observation of morphological changes in mouse liver and brain tissues. (**A**) Liver tissue, (**B**) brain tissue. BC: blank control group; AC: arecoline group.

Both the blank control group and the arecoline group exhibited intact liver structures with clear and complete lobular contours. The arrangement of the hepatic cords was orderly, with hepatic sinusoids distinctly discernible. No inflammatory cell infiltration or fibrous tissue proliferation was observed. The hepatocytes were large and plump, with polygonal or nearly round shapes, and the cytoplasm contained lipid droplets in varying sizes and numbers. There was no evidence of hepatocyte edema or necrosis. The nuclei were round and located in the center of the hepatocytes, with abundant chromatin, visible nucleoli, occasional binucleation, and no increase in mitotic figures. The liver interstitium showed no inflammatory cell infiltration (Figure 2A). In both the normal and the arecoline groups, the brain tissue cells within the field of view were structurally intact, with no necrosis or inflammatory infiltration observed. The pyramidal cells were arranged neatly, with clear cell borders and polygonal shapes (Figure 2B).

### 2.4. Effects of the Arecoline Solution on the Intestinal Microecosystem of Mice

#### 2.4.1. Effects of the Arecoline Solution on SCFAs in the Intestinal Contents of Mice

**Figure 3:**
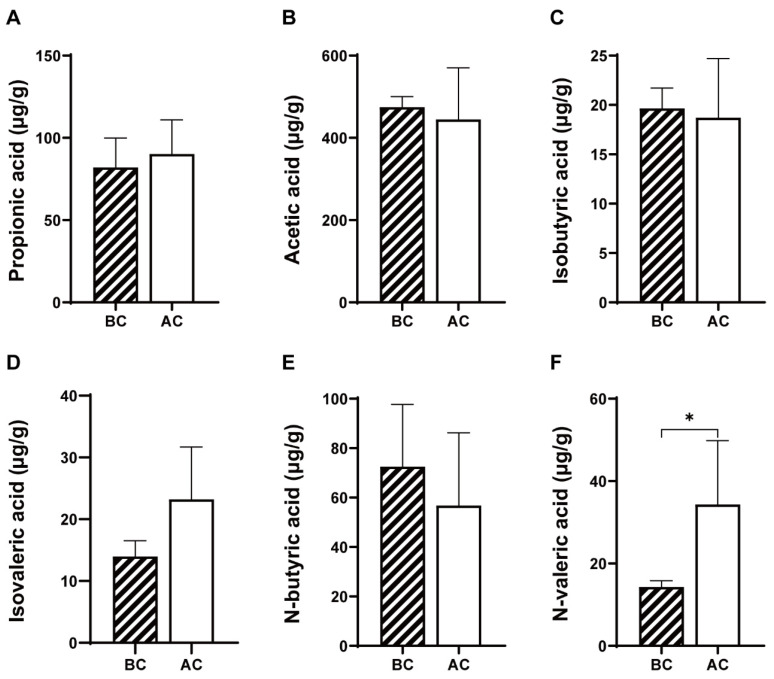
Effects of arecoline solution on short-chain fatty acids in mouse intestinal contents. (**A**) Propionic acid, (**B**) acetic acid, (**C**) isobutyric acid, (**D**) isovaleric acid, (**E**) N-butyric acid, (**F**) N-valeric acid. * *p* < 0.05. BC: blank control group; AC: arecoline group.

As shown in Figure 3, compared to the blank control group, the arecoline group exhibited increased levels of propionic acid and isovaleric acid and decreased levels of acetic acid, isobutyric acid, and n-butyric acid in the intestinal contents; however, these changes were not statistically significant (*p* > 0.05); the level of n-valeric acid was significantly increased (*p* < 0.05).

#### 2.4.2. Effects of the Arecoline Solution on Fecal Microbial Activity and ASVs in the Intestinal Mucosal Microbiota in Mice

**Figure 4:**
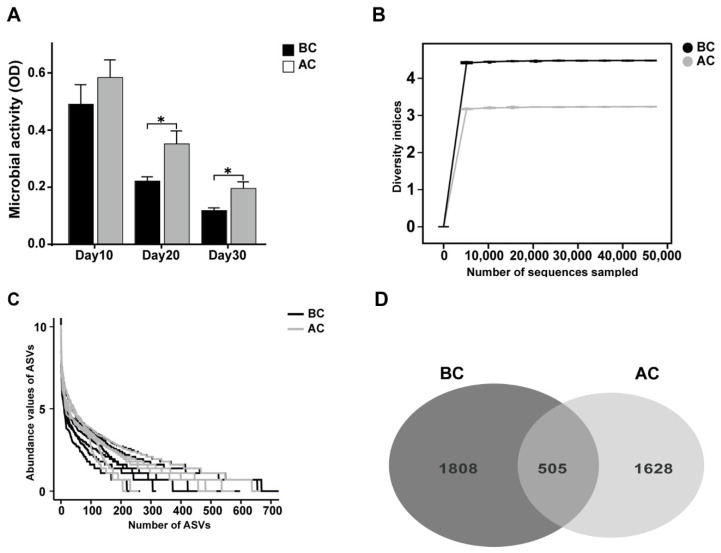
Effects of arecoline solution on fecal microbial activity and ASVs in intestinal mucosal microbiota in mice. (**A**) Fecal microbial activity, (**B**) rarefaction curve, (**C**) abundance rank curve, (**D**) Venn diagram based on the number of ASVs. * *p* < 0.05. BC: blank control group; AC: arecoline group.

Microbial activity is among the indices that gauge the collective metabolic capabilities of microorganisms within a community. As shown in Figure 4A, we dynamically monitored the microbial activity in the feces on days 10, 20, and 30 of the experiment. The results revealed that the intestinal microbial activity in the arecoline group was significantly higher than that in the blank control group on days 20 and 30 (*p* < 0.05). To delve deeper into the effects of the arecoline solution on the intestinal microbiota profile of the mice, we utilized 16S rRNA high-throughput sequencing technology to examine intestinal mucosa samples. As the sequencing depth of the samples increased, the rarefaction curves asymptotically approached a plateau, signifying that the current sequencing data adequately captured the species diversity within the samples (Figure 4B). The number of ASVs in the blank control group was 1808, while that in the arecoline group was 1628, with 505 ASVs shared between the two groups. This is consistent with the species abundance reflected by the abundance rank curve (Figure 4C,D).

#### 2.4.3. Effects of the Arecoline Solution on the Species Diversity of the Intestinal Mucosal Microbiota in Mice

**Figure 5:**
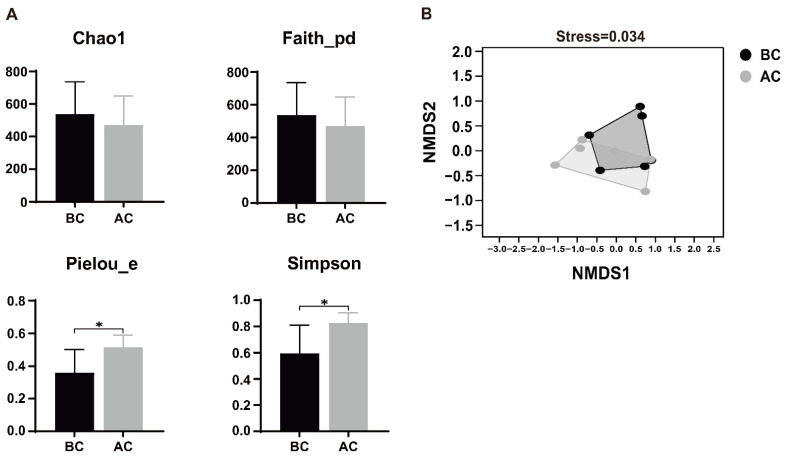
Effects of arecoline solution on species diversity in intestinal mucosal microbiota in mice. (**A**) Alpha diversity analysis, (**B**) NMDS analysis. * *p* < 0.05. BC: blank control group; AC: arecoline group.

Alpha diversity, a metric that quantifies species abundance, variety, and distribution uniformity within a uniformly sampled area, is alternatively termed intra-habitat diversity. The Chao1 index represents richness, the Simpson index represents species diversity, Faith’s PD index represents the diversity of species evolutionary relationships, and Pielou’s evenness index represents species evenness. As shown in Figure 5A, Pielou’s evenness index and Simpson index of the intestinal mucosal microbiota for the arecoline group were significantly higher than those for the blank control group (*p* < 0.05), indicating that the diversity and evenness of the intestinal mucosal microbiota in the examined mice underwent significant changes after administration of the arecoline solution. The beta diversity indices focus on the differences in species composition between communities. In this study, non-metric multidimensional scaling (NMDS) analysis revealed that there was some overlap in species between the two groups, but most species were discrete, indicating differences in the community structure of the intestinal mucosal microbiota between the two groups of mice. The stress value of the NMDS analysis result was 0.034, and a stress value less than 0.2 indicates that the results of an NMDS analysis are reasonable (Figure 5B).

#### 2.4.4. Effects of the Arecoline Solution on the Composition and Relative Abundance of the Intestinal Mucosal Microbiota in Mice

**Figure 6:**
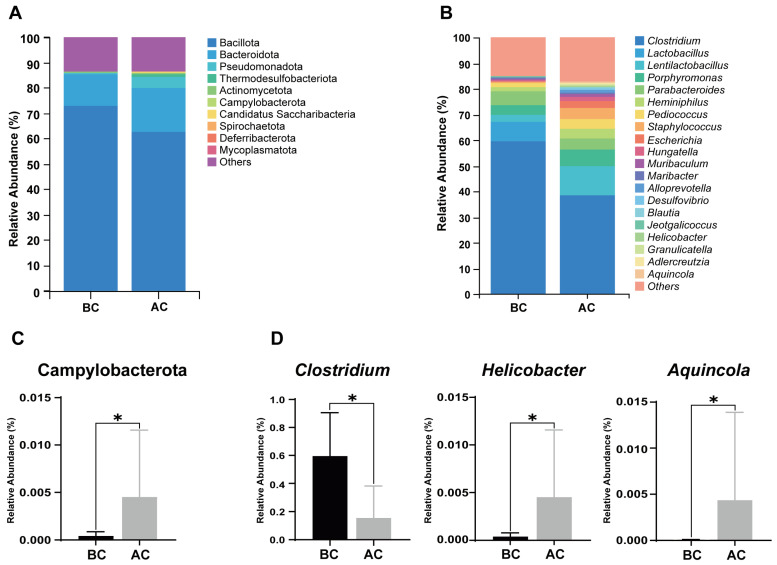
Influence of the arecoline solution on mouse intestinal mucosal microbiota composition and relative abundance. (**A**) Stacked bar chart of species composition and relative abundance at the phylum level, (**B**) stacked bar chart of species composition and relative abundance at the genus level, (**C**) differential phylum, (**D**) differential genera. * *p* < 0.05. BC: blank control group; AC: arecoline group.

A stacked bar chart of species composition is used to display the species composition across multiple samples. Figure 6A shows the relative abundance of bacteria in the mouse intestinal mucosa at the phylum level; as shown, Bacillota, Bacteroidota, Pseudomonadota, Thermodesulfobacteriota, Actinomycetota, Campylobacterota, Candidatus Saccharibacteria, Spirochaetota, Deferribacterota, and Mycoplasmatota are the top 10 dominant phyla, accounting for a significant proportion. Figure 6B illustrates the proportional abundance of bacteria in the mouse intestinal mucosal microbiota at the genus level, with *Clostridium*, *Lactobacillus*, and *Lentilactobacillus* being the dominant genera. Compared to the blank control group, at the phylum level, the relative abundance of Campylobacterota was increased in the arecoline group (Figure 6C, *p* < 0.05); at the genus level, the relative abundance of *Clostridium* was significantly decreased, while the relative abundances of *Helicobacter* and *Aquincola* were significantly increased in the intestinal mucosal microbiota of the mice in the arecoline group (Figure 6D, *p* < 0.05).

#### 2.4.5. Effects of the Arecoline Solution on Marker Species of the Intestinal Mucosal Microbiota in Mice

**Figure 7:**
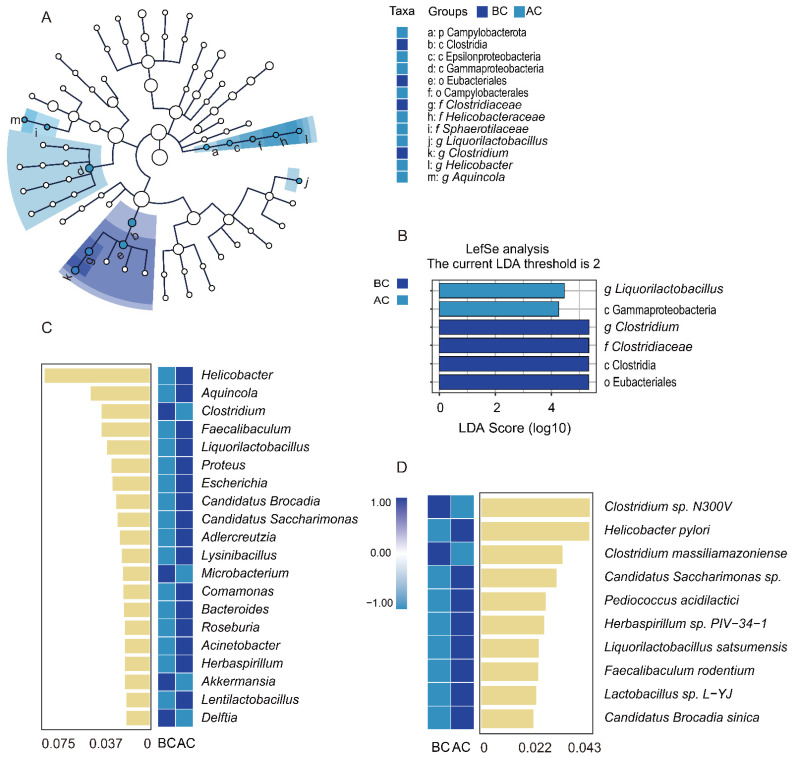
Impact of arecoline solution on distinct intestinal mucosa-associated microbiota profiles in mice. (**A**) Taxonomic cladogram based on LEfSe analysis, (**B**) bar plot showing species differences with an LDA threshold of 2, (**C**) random forest analysis at the genus level, (**D**) random forest analysis at the species level. BC: blank control group; AC: arecoline group.

Through linear discriminant analysis effect size (LefSe) analysis, we sought robust differential species among the sample groups. The LDA score distribution histogram presents the significantly enriched species within each group and their levels of importance, while the cladogram illustrates the taxonomic hierarchical distribution of the indicator species across the sample groups. In this study, with an LDA threshold set at 2, four indicator species were identified in the blank control group: Clostridia, Eubacteriales, *Clostridium*, and *Clostridiaceae*. In addition, two indicator species were identified in the arecoline group: *Liquorilactobacillus* and Gammaproteobacteria (Figure 7A,B). The results indicated significant differences in the characteristic intestinal mucosa-associated bacteria between the two groups. To identify the key species distinguishing the blank control group and the arecoline group, random forest diagnostic models were constructed at both the genus and the species levels, revealing nonlinear relationships among the variables. The heatmap displays the abundance distribution of these species across the groups, with the importance of the species in the model decreasing from top to bottom. These top-ranked species are indicator species for the inter-group differences. As shown in Figure 7C, at the genus level, *Clostridium*, *Microbacterium*, *Akkermansia*, and *Delftia* were the indicator species for the blank control group. *Helicobacter*, *Aquincola*, *Faecalibaculum*, and *Liquorilactobacillus* were the top four indicator species for the arecoline group. As shown in Figure 7D, at the species level, *Clostridium* sp. *N300V* and *Clostridium massiliamazoniense* were the indicator species for the blank control group, while *Helicobacter pylori* and *Candidatus Saccharimonas* sp. were the top two indicator species for the arecoline group.

### 2.5. Correlation Analysis Between GABA, DA, 5-HT, and BDNF in Brain Tissue and the Intestinal Mucosal Microbiota

**Figure 8:**
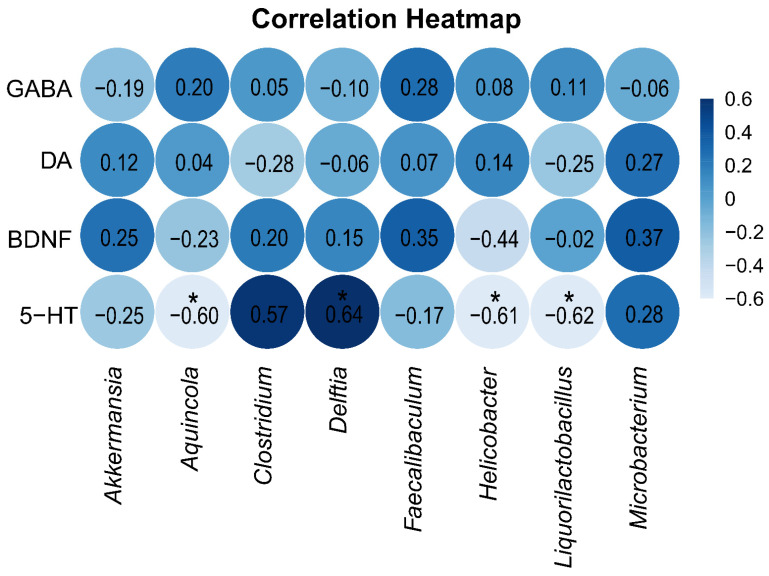
Spearman correlation analysis heatmap. A deeper blue color signifies a stronger positive correlation between the parameters, whereas a lighter blue indicates a stronger negative correlation. * *p* < 0.05.

To explore the relationship between neurotransmitters and neurotrophic factors in brain tissue on one hand and the intestinal mucosa microbiota on the other, Spearman correlation analysis was performed between the indicator species of the two groups and the levels of GABA, DA, 5-HT, and BDNF in brain tissue. The correlation heatmap (Figure 8) shows that the level of 5-HT in brain tissue was significantly positively correlated with the indicator genus Delftia in the blank control group and significantly negatively correlated with the indicator genera Aquincola, Helicobacter, and Liquorilactobacillus in the arecoline group.

## 3. Discussion

### 3.1. Arecoline Alters Mouse Behavior and Exerts Significant Effects on DA and 5-HT

Arecoline, a psychoactive alkaloid, functions as a stimulant for both the central and the autonomic nervous systems by modulating neurotransmitter activity, thereby augmenting central nervous system stimulation, alertness, and attention [23]. To evaluate anxiety, exploratory behavior, and locomotor activity in animals, particularly rodents like mice and rats, researchers frequently employ the open-field test. This test involves measuring various parameters, including total distance covered, mean velocity, duration of rapid movement, and frequency of central zone entries. These metrics collectively assess an animal’s locomotor activity and overall activity levels [24]. In the open-field test, it was observed that the mice in the arecoline group showed a significant increase in the number of entries into the central area and the time spent in fast movement on day 21 of the experiment, with the number of entries into the central area still significantly higher than that of the blank control group on day 28. However, no significant differences were observed in total distance traveled and average speed. These findings suggest that, under the experimental conditions, the dose of arecoline and the duration of arecoline administration were effective in producing its stimulating effects, enhancing the exploratory behavior and activity to some extent in mice.

To further investigate the effects of the arecoline solution on neurotransmitters and neurotrophic factors in mice, this study measured the levels of GABA, DA, 5-HT, and BDNF in brain and liver tissues. The findings revealed that, in comparison to the blank control group, the arecoline group exhibited significantly decreased concentrations of DA in the liver and 5-HT in the brain. DA, a pivotal catecholaminergic neurotransmitter in the mammalian central nervous system, plays a crucial role in regulating neural functions, including motor activity, coordination, mood, and endocrine balance [25]. Furthermore, DA is also found in peripheral tissues, where it modulates metabolic processes by influencing adenosine 5′-monophosphate (AMP)-activated protein kinase (AMPK) phosphorylation in the liver, adipose tissue, and skeletal muscle and regulates proteins associated with lipid metabolism [26]. Research has demonstrated that DA exhibits anti-inflammatory properties in both acute and chronic inflammatory conditions by inhibiting the differentiation of adaptive immune cells and fostering immune homeostasis [27]. In our investigation, we did not observe any notable alterations in the DA concentrations within the brain tissue, suggesting that the experimental dosage and duration of arecoline administration did not elicit changes in the central nervous system DA levels. However, the significant decrease in DA found in the liver implies potential effects of arecoline on liver metabolic processes or immune balance. 5-HT, a monoamine synthesized from tryptophan through two steps involving tryptophan hydroxylase (TPH) and aromatic L-amino acid decarboxylase (AADC), not only functions as a neurotransmitter but also exhibits hormonal, autocrine, and paracrine activities [28]. It is involved in numerous physiological processes in the human body, contributing to learning, sleep, memory, emotion, and other aspects [29]. The 5-HT system exerts intricate regulatory influences on cognitive and motor functions [30]. Studies have shown that the level of 5-HT in the brain of mice with central fatigue is significantly increased, and ginseng and Korean red ginseng can significantly regulate 5-HT activity in the brain, reducing the 5-HT levels and exerting anti-central fatigue effects [31]. In the present study, it was found that the level of 5-HT in the brain of the mice in the arecoline group was decreased, which may be a mechanism underlying the stimulating effects of arecoline. However, it is important to note that 5-HT interacts with a diverse and intricate array of central nervous system receptors [32], and its mechanisms of action are influenced by multiple factors.

### 3.2. Arecoline Enhances Intestinal Microbial Activity and Affects the SCFA Levels in the Intestine

As the significance of the brain–gut axis garners increasing attention, this study also focused on investigating the impact of the arecoline solution on the intestinal microecosystem in mice. The dynamic monitoring of microbial activity in mouse feces revealed a significant increase in fecal microbial activity on days 20 and 30 of the experiment in the arecoline group. Microbial activity is a key indicator reflecting and assessing the metabolic function of microorganisms within a community [33]. These findings suggest that administering arecoline can elevate the level of active microorganisms in mouse feces, although the precise timing of this occurrence remains uncertain and may be even earlier than that indicated. The enhancement of active microorganisms could potentially influence physiological functions mediated by the microbiota, such as food digestion, metabolism, and immune regulation. SCFAs, which are primarily produced in the intestinal tract through microbial fermentation of dietary fiber, exhibit a range of physiological effects, including immune modulation and anti-inflammatory actions [34]. Presently, accumulating evidence underscores the extensive influence of SCFAs on pivotal neurological and behavioral processes. SCFAs are capable of regulating neurotransmitter and neurotrophic factor levels, thereby playing a fundamental role in brain development and the preservation of central nervous system homeostasis [35]. In the present study, it was found that the mice in the arecoline group exhibited increased levels of n-valeric acid, isovaleric acid, and propionic acid, along with decreased levels of acetic acid, isobutyric acid, and n-butyric acid in their intestinal contents, with a significant increase observed in n-valeric acid levels (*p* < 0.05). n-Valeric acid functions as an immunometabolic modulator, capable of suppressing the differentiation of Th17 cells in the small intestine and alleviating the inflammation in the central nervous system induced by segmented filamentous bacterial colonization [36]. Studies have shown that improvements in symptoms related to emotional stress are accompanied by elevated concentrations of n-valeric acid in the feces [37]. Despite the fact that the exact mechanisms by which SCFAs exert their effects on the central nervous system remain largely elusive, our experimental findings hint at a potential association between n-valeric acid and the stimulating effect of arecoline.

### 3.3. Arecoline Alters the Structure and Diversity of the Intestinal Mucosal Microbiota in Mice

We observed that the arecoline solution intervention exerted a significant impact on the diversity, composition, and characteristics of the intestinal mucosal microbiota in mice. Compared to the blank control group, the Pielou_e index and Simpson index of the intestinal mucosal microbiota were significantly elevated for the arecoline group, indicating that the arecoline solution influenced the diversity and evenness of the intestinal mucosal microbiota. The relative abundance of Campylobacterota was increased in the arecoline group. Campylobacterota is a predominantly Gram-negative, spiral-shaped bacterial group whose components share a common energy-conserving mechanism across diverse microenvironments [38]. These bacteria establish ion gradients (proton or sodium gradients) through redox reactions, driving flagellar rotation and ATP production, a process crucial for the transport of chemicals across the membrane. Their polar flagella afford them high motility and act as micro-mixers, thereby facilitating the continual influx of nutrients into their microenvironment. By utilizing chemotaxis, Campylobacterota are able to navigate towards more conducive growth conditions [39]. Investigations have shown that in mice subjected to chronic stress, there is an elevation in *Campylobacter* abundance at the family, order, class, and genus levels, with a significant correlation observed between the host’s metabolism of D-glutamate and D-glutamine and alterations in *Campylobacter* populations [40]. Glutamate is one of the major neurotransmitters in the central nervous system. At the genus level, the relative abundance of *Clostridium* in the intestinal mucosal microbiota of mice in the arecoline group was significantly decreased, while the relative abundances of *Helicobacter* and *Aquincola* were significantly increased. Both LEfSe analysis and random forest analysis identified *Liquorilactobacillus* as a biomarker species in the arecoline group. *Liquorilactobacillus,* a lactic acid bacterium, holds the potential to modulate the production of metabolites that are beneficial to health, thereby exerting favorable effects on the host [41,42]. The genus *Clostridium* encompasses a diverse group of Gram-positive, anaerobic, endospore-forming bacteria, predominantly non-pathogenic, though a minority possesses pathogenic traits [43]. The primary physiological functions of *Clostridium* encompass direct interaction with the immune system and the generation of SCFAs, which serve crucial regulatory roles in maintaining intestinal and immune homeostasis in the body [44].

*Aquincola* is a Gram-negative, sulfate-reducing bacterium capable of metabolizing through sulfate reduction in anaerobic conditions, thriving particularly in environments abundant in sulfides and organic matter. Its association with human diseases remains unclear [45]. *Helicobacter*, characterized by its Gram-negative status, spiral shape, and microaerophilic nature, primarily populates the stomach and intestine [46]. Notably, *Helicobacter pylori* (*H. pylori*) stands out as the most prominent species within this genus. *H. pylori* possesses an array of virulence factors and enzymes that allow it to endure the highly acidic gastric environment. Outer-membrane proteins such as BabA, SabA, OipA, and HopQ play crucial roles in bacterial colonization by mediating the adhesion of *H. pylori* to gastric epithelial cells, thereby promoting persistent infection. This chronic infection can span a spectrum of diseases, ranging from gastritis and peptic ulcer disease to more severe conditions such as mucosa-associated lymphoid tissue lymphoma and non-cardia gastric cancer [47,48]. Therefore, the finding from the random forest analysis at the species level that *H. pylori* was a biomarker species in the arecoline group cannot but alert us to the potential health issues that arecoline may pose.

### 3.4. Correlation Between the 5-HT Levels in Brain Tissue and Marker Bacterial Genera of the Intestinal Mucosal Microbiota

Based on the correlation heatmap analysis, we found a significant positive correlation between the 5-HT levels in brain tissue and the abundance of the signature genus *Delftia* in the blank control group, indicating that the *Delftia* genus may be involved in or influence the synthesis or regulatory mechanisms of 5-HT, thereby affecting the host’s neural function and behavioral outcomes through this pathway. In contrast, we observed a significant negative correlation between the 5-HT levels and the signature genera *Aquincola*, *Helicobacter*, and *Liquorilactobacillus* in the arecoline group. This finding suggests that these signature genera may impact the host’s 5-HT levels by inhibiting 5-HT synthesis or promoting its degradation. Notably, *Helicobacter*, which is known to be associated with gastrointestinal diseases, showed an increased abundance in the arecoline group, which potentially points to more complex interactions that not only affect gastrointestinal health but also may interfere with the critical role of the intestinal microbiota in regulating the host 5-HT.

## 4. Material and Methods

### 4.1. Preparation of the Experimental Animals

To mitigate the potential impact of gender on the mouse intestinal microbiota [49], male SPF-grade Kunming (KM) mice weighing 20 ± 2 g were chosen and sourced from Hunan Slaccas Laboratory Animal Co., Ltd. (Changsha, China). These mice were housed within an SPF-grade-barrier environment at the Animal Experiment Center of Hunan University of Chinese Medicine, maintained at a room temperature of 23–25 °C with a relative humidity range of 50–70% and subjected to a controlled 12 h light/dark cycle. The mice had unrestricted access to a standard pellet diet and water. This study was ethically reviewed and approved by the Institutional Animal Care and Use Committee of Hunan University of Chinese Medicine, under approval number HNUCM-21-2312-09.

### 4.2. Information on Animal Feed

The mice were fed a growth and reproduction diet for experimental mice irradiated with Co60. This product has moisture content ≤ 100 g, crude protein content ≥ 200 g, crude fiber content ≥ 40 g, crude fat content ≤ 50 g, crude ash content ≤ 80 g, calcium content of 10–18 g, phosphorus content of 6–12 g, with a calcium-to-phosphorus ratio between 1.2:1 and 1.7:1, lysine content ≥ 13.2 g, and total content of methionine and cysteine ≥ 7.8 g. This diet was sourced from the Animal Experiment Center of Hunan University of Chinese Medicine and manufactured by Jiangsu Meidi Biopharmaceutical Co., Ltd. (Yangzhou, China), under license number SYXK (Hunan) 2020-0006.

### 4.3. Pharmaceuticals and Kits

Arecoline (S26HB195898) was purchased from Shanghai Yuanye Biotech Co., Ltd. (Shanghai, China) GABA (JM-02725M2), DA (JM-02906M2), 5-HT (JM-02726M2), and brain-derived neurotrophic factor (BDNF, JM-02487M2) ELISA kits were purchased from Jiangsu Jingmei Biotech Co., Ltd. (Yancheng, China).

### 4.4. Animal Grouping and Drug Administration

After three days of acclimation feeding, twenty KM mice were randomly divided into two groups: a blank control group (BC) and an arecoline group (AC). The arecoline dosage was based on the average daily intake of arecoline in adults and was kept within the range of acute toxicity doses [8]. The mice in the arecoline group received 2 mg/(kg·d) of an arecoline solution administered via gavage twice daily, with each dose volume being 0.3 mL, for 30 consecutive days. The mice in the blank control group received an equal volume of sterile water via gavage twice daily, 0.3 mL each time, for 30 consecutive days.

### 4.5. Open-Field Test

Open-field tests were conducted on days 7, 14, 21, and 28 of the experiment. A pillar-shaped open-field box with blue walls and bottom was used. The bottom of the box was divided into 16 equal squares. The mice were positioned at the center of the box’s bottom. Following a 1 min adaptation period, a SMART real-time video tracking and analysis system was employed to record the distance traveled, average speed, time spent in fast movement, and number of entries into the center by the mice over a 6 min period. In addition, the experiments took place in a dimly lit and tranquil environment to minimize external distractions. After each mouse was tested, excretions were thoroughly removed, and the box was thoroughly wiped with alcohol to eliminate any odors and ensure a consistent testing environment. After the experiment, the results were analyzed using video analysis software.

### 4.6. Sample Collection

On days 10, 20, and 30 of the experiment, fresh feces were collected from the mice in a relatively sterile environment. At the conclusion of the experiment, the mice were euthanized via cervical dislocation, and their livers and brain tissues were harvested. In relatively sterile conditions, sterile forceps were used to collect the intestinal contents from the jejunum to the ileum. Subsequently, a sterile coverslip was employed to gently scrape off the intestinal mucosa to obtain samples. These collected intestinal content and mucosa samples were then individually stored in separate EP tubes at −80 °C for subsequent analysis [50].

### 4.7. Detection of GABA, DA, 5-HT, and BDNF in Liver and Brain Tissues

The liver and brain tissues were mixed with physiological saline and steel beads in a specified ratio. Following homogenization using a tissue grinder, the mixture was centrifuged at 3000 rpm for 10 min at 4 °C using a low-temperature centrifuge. The supernatant was then collected and stored at low temperature for subsequent use [51,52]. The concentrations of GABA, DA, 5-HT, and BDNF in the mouse liver and brain tissues were determined using the ELISA method. Sample addition, incubation, plate washing, and detection were carried out according to the manufacturer’s instructions, and the concentrations were calculated based on optical density (OD) values.

### 4.8. Histological Analysis of Liver and Brain Tissues

The liver and brain tissues from the mice were processed through routine dehydration, immersion, embedding, sectioning, and staining with hematoxylin and eosin (HE). Subsequently, morphological alterations in these tissues were examined under an optical microscope [53].

### 4.9. Fecal Microbial Activity Assay

Sterile water was added to the fecal samples in a ratio of 50 mL of sterile water for every 3 g of feces, and the mixture was shaken for 30 min to fully extract an enzymatic solution from the samples. The mixture was then centrifuged at 3000 rpm for 10 min using a low-temperature centrifuge, and the collected supernatant was the crude enzyme solution. Microbial activity in the fecal samples was determined using the fluorescein diacetate (FDA) hydrolysis method. For each group, one blank tube and three sample tubes were prepared. For the blank tube, 2 mL of the FDA reaction solution, 2 mL of acetone, and 50 µL of sample were added to a dry sterilized test tube, and the mixture was incubated at 24 °C for 90 min. For the sample tubes, 2 mL of the FDA reaction solution and 50 µL of sample were added to a dry sterilized test tube, and the mixture was incubated at 24 °C for 90 min before adding 2 mL of acetone to stop the reaction. Finally, the absorbance was assessed at 490 nm with a UV spectrophotometer, enabling the calculation of microbial activity per unit mass of the sample [54,55].

### 4.10. Detection of SCFAs in the Intestinal Content

In this experiment, intestinal contents were collected for a quantitative analysis of short-chain fatty acids, and the analysis was conducted by Qingdao Yixin Testing Technology Service Co., Ltd. (Qingdao, China). The concentrations of propionic acid, acetic acid, isobutyric acid, isovaleric acid, n-butyric acid, and n-valeric acid in the samples were ascertained utilizing gas chromatography–mass spectrometry (GC-MS) analysis. The GC-MS conditions are presented in Table 2. A stock solution was prepared by dissolving 0.125 g of propionic acid, acetic acid, isobutyric acid, isovaleric acid, n-butyric acid, and n-valeric acid in 100 mL of ether. After preparation, the stock solution was added to the samples for analysis, and a standard curve was established. For sample preparation, 2 mL of a 1:3 phosphate-buffered saline solution was added to the sample, and the mixture was vortexed and homogenized for 2 min. Then, 1 mL of ether was added for extraction for 10 min, followed by centrifugation at 4000 rpm for 20 min. Subsequently, 1 mL of the short-chain fatty acid stock solution was added, and the mixture was centrifuged at 4000 rpm for 10 min to ensure complete mixing and volatilization of the two extracts. Finally, the concentrations of propionic acid, acetic acid, isobutyric acid, isovaleric acid, n-butyric acid, and n-valeric acid in the intestinal contents of the mice were measured using the above-mentioned instrument.

### 4.11. DNA Extraction, 16S rRNA Gene Amplicon Sequencing, and Sequence Analysis

The small intestinal mucosa samples were pretreated, and nucleic acids were extracted using the OMEGA Soil DNA Kit (D5635-02) (Omega Bio-Tek, Norcross, GA, USA). The extracted DNA was subjected to 0.8% agarose gel electrophoresis to assess its molecular size, and quantified using a Nanodrop (Thermo Scientific, NC2000, NY, USA). For this project, the highly variable V3V4 region of the bacterial 16S rRNA gene was selected for sequencing. PCR amplification of the bacterial 16S rRNA gene was performed using the forward primer 338F (5′-ACTCCTACGGGAGGCAGCA-3′) and the reverse primer 806R (5′-GGACTACHVGGGTWTCTAAT-3′). The PCR products were quantified on a Microplate reader (BioTek, FLX800T, VT, USA) using the Quant-iT PicoGreen dsDNA Assay Kit (Thermo Scientific, P7589, CA, USA) and then pooled according to the required data volume for each sample. Library preparation was carried out using the TruSeq Nano DNA LT Library Prep Kit from Illumina. The libraries were quantified on a Promega QuantiFluor (Promega Corporation, dsDNA System, WI, USA) using the Quant-iT PicoGreen dsDNA Assay Kit (Thermo Scientific, P7589, CA, USA), with acceptable libraries having a concentration of 2 nM or above. Qualified libraries were subjected to paired-end sequencing on either the Illumina NovaSeq (Illumina, PE250, CA, USA) platform. Sequencing was performed by Shanghai Personal Biotechnology Co., Ltd. (Shanghai, China).

### 4.12. Bioinformatics Analysis of High-Throughput Sequencing Data

The raw sequencing data underwent sequence denoising to yield ASVs, whose representative sequences were matched against reference sequences in the SILVA database to ascertain corresponding taxonomic information [56].

(1) Alpha diversity: Alpha diversity refers to indices measuring species richness and diversity within a localized, homogeneous habitat. Indices like Chao1, Observed_species, Shannon, and Simpson were analyzed through QIIME2 (https://qiime2.org/; accessed on 4 November 2024).

(2) Beta diversity: Beta diversity is utilized to investigate variations in species composition across temporal and spatial gradients. We computed four distance matrices: Jaccard, Bray–Curtis, unweighted UniFrac, and weighted UniFrac. These distance matrices were then subjected to NMDS analysis, and visualization was achieved in QIIME2 (https://view.qiime2.org/; accessed on 4 November 2024).

(3) LEfSe analysis: LEfSe analysis (http://huttenhower.sph.harvard.edu/lefse/; accessed on 4 November 2024) is a method that combines non-parametric Kruskal–Wallis and Wilcoxon rank-sum tests with linear discriminant analysis (LDA) effect size. LEfSe analysis allows for a simultaneous differential analysis across all taxonomic levels. Additionally, LEfSe emphasizes the identification of robust differential species, i.e., biomarker species, between groups.

### 4.13. Statistical Analysis

Statistical analyses were performed using IBM SPSS Statistics version 25.0 (IBM Corp., Armonk, NY, USA). For quantitative data that followed a normal distribution, comparisons between two groups were conducted using an independent sample *t*-test. If the data did not follow a normal distribution, non-parametric tests were used. A *p*-value < 0.05 was considered statistically significant.

## 5. Conclusions

Under the effect of arecoline, mice exhibited significant behavioral changes and alterations in the 5-HT levels in the brain, leading to notable excitement and enhanced locomotor activity. Arecoline markedly increased microbial activity in mouse feces and elevated the level of n-valeric acid in the intestinal content, significantly impacting the structure, composition, and diversity of the intestinal mucosal microbiota. A significant negative correlation was observed between the 5-HT levels and the abundance of the indicator genera *Aquincola*, *Helicobacter*, and *Liquorilactobacillus*. These findings suggest that arecoline exerts a notable influence on the microecosystem in multi-ecological intestinal sites and that there is an association between changes in the intestinal microbiota and the 5-HT levels, which may constitute one of the key mechanisms underlying the stimulating effects of arecoline. However, the specific long-term effects of the arecoline solution on organismal health, whether positive or negative, remain unclear. Therefore, further research is warranted in the future to validate these findings.

## Figures and Tables

**Table 1:** Statistical table of spontaneous activity in mice.

		Total Distance Traveled	Average Speed	Time Spent in Fast Movement	Number of Entries into the Center
Day 7	BC	1588.07 ± 320.74	4.41 ± 0.89	7.70 ± 3.55	18.80 ± 6.57
	AC	1634.35 ± 357.02	4.54 ± 0.99	6.45 ± 3.50	18.60 ± 4.16
Day 14	BC	1195.87 ± 104.94	3.32 ± 0.29	5.19 ± 1.89	7.80 ± 4.32
	AC	1299.96 ± 252.78	3.61 ± 0.70	6.21 ± 3.15	6.00 ± 3.32
Day 21	BC	1195.87 ± 104.94	3.32 ± 0.29	4.48 ± 1.12	7.40 ± 1.14
	AC	1299.96 ± 252.78	3.61 ± 0.70	8.30 ± 3.12 *	14.00 ± 1.41 *
Day 28	BC	1002.55 ± 398.32	2.78 ± 1.11	4.48 ± 2.26	3.80 ± 0.45
	AC	977.68 ± 166.76	2.72 ± 0.47	5.28 ± 1.34	8.60 ± 2.30 *

Note: values are presented as mean ± standard deviation (*n* = 5). * *p* < 0.05. BC: blank control group; AC: arecoline group.

**Table 2:** GC-MS conditions.

Steps	Conditions
**Column temperature requirement**	Initial Temperature: 100 °C, held for 5 min Ramp to 150 °C at 5 °C/min (no hold) Immediate Ramp to 240 °C at 30 °C/min Final Temperature: 240 °C, held for 30 min
**Flow rate requirement**	1 mL/min
**Shunt ratio**	75:1
**Carrier gas**	Helium
**Chromatographic column**	TG WAX 30 m × 0.25 mm × 0.25 μm
**Injector**	240 °C
**Bombardment voltage**	70 eV
**Single-ion scan mode**	Quantitative ion 63, 70
**Ion source temperature**	200 °C
**Connection line temperature**	250 °C

## Data Availability

The data underlying this study are available within the manuscript. The intestinal mucosal microbiota sequencing data have been uploaded to the NCBI database (https://www.ncbi.nlm.nih.gov/; accessed on 1 January 2025), NO. PRJNA1205108.

## References

[1] Chatterjee N., Gupte H.A. (2023). Areca nut use among adolescents: How do we prevent and control this problem?. J. Glob. Health.

[2] Benegal V., Rajkumar R.P., Muralidharan K. (2008). Does areca nut use lead to dependence?. Drug Alcohol. Depend..

[3] Oliveira N.G., Ramos D.L., Dinis-Oliveira R.J. (2021). Genetic toxicology and toxicokinetics of arecoline and related areca nut compounds: An updated review. Arch. Toxicol..

[4] Ansari A., Mahmood T., Bagga P., Shamim A., Ahmad S. (2021). Areca catechu: A phytopharmacological legwork. Food Front..

[5] Dangore-Khasbage S., Bhowate R.R., Khubchandani M. (2023). Chemical Composition of Areca Nut and Its Adverse Effects on Human Health. Cureus.

[6] Pfeiffer C.C., Beck R.A., Goldstein L. (1967). The modification of central nervous system (CNS) function by autonomic drugs. Amine shift responses differentiate be-tween CNS nicotinic and nuscarinic effects. Ann. N. Y. Acad. Sci..

[7] Volgin A.D., Bashirzade A., Amstislavskaya T.G., Yakovlev O.A., Demin K.A., Ho Y.J., Wang D., Shevyrin V.A., Yan D., Tang Z. (2019). DARK classics in chemical neuroscience: Arecoline. ACS Chem. Neurosci..

[8] Xiao B., Xiao N., Peng M., Gong L., Li C., Lin L. (2013). Research on effects of arecoline on refreshing and acute toxicity test. China Mod. Med..

[9] Arias-Carrión O., Caraza-Santiago X., Salgado-Licona S., Salama M., Machado S., Nardi A.E., Menéndez-González M., Murillo-Rodríguez E. (2014). Orquestic regulation of neurotransmitters on reward-seeking behavior. Int. Arch. Med..

[10] Begum N., Mandhare A., Tryphena K.P., Srivastava S., Shaikh M.F., Singh S.B., Khatri D.K. (2022). Epigenetics in depression and gut-brain axis: A molecular crosstalk. Front. Aging Neurosci..

[11] Tao R., Liu S., Crawford J., Tao F. (2023). Gut-Brain Crosstalk and the Central Mechanisms of Orofacial Pain. Brain. Sci..

[12] Mahbub N.U., Islam M.M., Hong S.T., Chung H.J. (2024). Dysbiosis of the gut microbiota and its effect on α-synuclein and prion protein misfolding: Consequences for neurodegeneration. Front. Cell. Infect. Microbiol..

[13] Li Y., Luo Z.Y., Hu Y.Y., Bi Y.W., Yang J.M., Zou W.J., Song Y.L., Li S., Shen T., Li S.J. (2020). The gut microbiota regulates autism-like behavior by mediating vitamin B6 homeostasis in EphB6-deficient mice. Microbiome.

[14] Dohnalová L., Lundgren P., Carty J.R.E., Goldstein N., Wenski S.L., Nanudorn P., Thiengmag S., Huang K.P., Litichevskiy L., Descamps H.C. (2022). A microbiome-dependent gut-brain pathway regulates motivation for exercise. Nature.

[15] Vafadari B. (2021). Stress and the role of the gut-brain axis in the pathogenesis of schizophrenia: A Literature review. Int. J. Mol. Sci..

[16] Yano J.M., Yu K., Donaldson G.P., Shastri G.G., Ann P., Ma L., Nagler C.R., Ismagilov R.F., Mazmanian S.K., Hsiao E.Y. (2015). Indigenous bacteria from the gut microbiota regulate host serotonin biosynthesis. Cell.

[17] Zhou M., Fan Y., Xu L., Yu Z., Wang S., Xu H., Zhang J., Zhang L., Liu W., Wu L. (2023). Microbiome and tryptophan metabolomics analysis in adolescent depression: Roles of the gut microbiota in the regulation of tryptophan-derived neurotransmitters and behaviors in human and mice. Microbiome.

[18] Zhuang Z., Yang R., Wang W., Qi L., Huang T. (2020). Associations between gut microbiota and Alzheimer’s disease, major depressive disorder, and schizophrenia. J. Neuroinflammation.

[19] Ketchesin K.D., Becker-Krail D., McClung C.A. (2020). Mood-related central and peripheral clocks. Eur. J. Neurosci..

[20] Hirayama M., Mure L.S., Le H.D., Panda S. (2024). Neuronal reprogramming of mouse and human fibroblasts using transcription factors involved in suprachiasmatic nucleus development. iScience.

[21] Bravo J.A., Forsythe P., Chew M.V., Escaravage E., Savignac H.M., Dinan T.G., Bienenstock J., Cryan J.F. (2011). Ingestion of Lactobacillus strain regulates emotional behavior and central GABA receptor expression in a mouse via the vagus nerve. Proc. Natl. Acad. Sci. USA.

[22] Konturek T.J., Martinez C., Niesler B., van der Voort I., Mönnikes H., Stengel A., Goebel-Stengel M. (2021). The role of brain-derived neurotrophic factor in irritable bowel syndrome. Front. Psychiatry.

[23] Ragab A.E., Badawy E.T., Aboukhatwa S.M., Kabbash A., Abo El-Seoud K.A. (2022). In vitro characterization of inhibitors for lung A549 and leukemia K562 cell lines from fungal transformation of arecoline supported by in silico docking to M3-mAChR and ADME prediction. Pharmaceuticals.

[24] Paydar A., Lee B., Gangadharan G., Lee S., Hwang E.M., Shin H.S. (2014). Extrasynaptic GABAA receptors in mediodorsal thalamic nucleus modulate fear extinction learning. Mol. Brain.

[25] Neumann J., Hofmann B., Dhein S., Gergs U. (2023). Role of Dopamine in the Heart in Health and Disease. Int. J. Mol. Sci..

[26] Tavares G., Martins F.O., Melo B.F., Matafome P., Conde S.V. (2021). Peripheral Dopamine Directly Acts on Insulin-Sensitive Tissues to Regulate Insulin Signaling and Metabolic Function. Front. Pharmacol..

[27] Li M., Zhou L., Sun X., Yang Y., Zhang C., Wang T., Fu F. (2022). Dopamine, a co-regulatory component, bridges the central nervous system and the immune system. Biomed. Pharmacother..

[28] Császár-Nagy N., Bob P., Bókkon I. (2022). A Multidisciplinary Hypothesis about Serotonergic Psychedelics. Is it Possible that a Portion of Brain Serotonin Comes from the Gut?. J. Integr. Neurosci..

[29] Neumann J., Hofmann B., Dhein S., Gergs U. (2023). Cardiac Roles of Serotonin (5-HT) and 5-HT-Receptors in Health and Disease. Int. J. Mol. Sci..

[30] Flaive A., Fougère M., van der Zouwen C.I., Ryczko D. (2020). Serotonergic Modulation of Locomotor Activity from Basal Vertebrates to Mammals. Front. Neural. Circuits.

[31] Kang J.Y., Kim D.Y., Lee J.S., Hwang S.J., Kim G.H., Hyun S.H., Son C.G. (2021). Korean Red Ginseng Ameliorates Fatigue via Modulation of 5-HT and Corticosterone in a Sleep-Deprived Mouse Model. Nutrients..

[32] Meeter M., Talamini L., Schmitt J.A., Riedel W.J. (2006). Effects of 5-HT on memory and the hippocampus: Model and data. Neuropsychopharmacology.

[33] Zhou M., Li X., Liu J., Wu Y., Tan Z., Deng N. (2024). Adenine’s impact on mice’s gut and kidney varies with the dosage administered and relates to intestinal microorganisms and enzyme activities. 3 Biotech.

[34] Rasouli-Saravani A., Jahankhani K., Moradi S., Gorgani M., Shafaghat Z., Mirsanei Z., Mehmandar A., Mirzaei R. (2023). Role of microbiota short-chain fatty acids in the pathogenesis of autoimmune diseases. Biomed. Pharmacother..

[35] Silva Y.P., Bernardi A., Frozza R.L. (2020). The Role of Short-Chain Fatty Acids from Gut Microbiota in Gut-Brain Communication. Front. Endocrinol..

[36] Luu M., Pautz S., Kohl V., Singh R., Romero R., Lucas S., Hofmann J., Raifer H., Vachharajani N., Carrascosa L.C. (2019). The short-chain fatty acid pentanoate suppresses autoimmunity by modulating the metabolic-epigenetic crosstalk in lymphocytes. Nat. Commun..

[37] Nishida K., Sawada D., Kuwano Y., Tanaka H., Rokutan K. (2019). Health Benefits of *Lactobacillus gasseri* CP2305 Tablets in Young Adults Exposed to Chronic Stress: A Randomized, Double-Blind, Placebo-Controlled Study. Nutrients.

[38] Yang T., Chen S., Qiu L., Guo Q., Wang Z., Jiang Y., Bai H., Bi Y., Chang G. (2024). Effect of high dietary iron on fat deposition and gut microbiota in chickens. Animals.

[39] van der Stel A.X., Wösten M.M.S.M. (2019). Regulation of respiratory pathways in campylobacterota: A review. Front. Microbiol..

[40] Zhang B., Dong W., Ma Z., Duan S., Han R., Lv Z., Liu X., Mao Y. (2023). Hyperbaric oxygen improves depression-like behaviors in chronic stress model mice by remodeling gut microbiota and regulating host metabolism. CNS Neurosci. Ther..

[41] De Filippis F., Pasolli E., Ercolini D. (2020). The food-gut axis: Lactic acid bacteria and their link to food, the gut microbiome and human health. FEMS Microbiol. Rev..

[42] Larini I., Tintori S., Gatto V., Felis G.E., Salvetti E., Torriani S. (2024). Comparative genomics reveals the potential biotechnological applications of *Liquorilactobacillus nagelii* VUCC-R001, a strain isolated from kombucha tea. Food Biosci..

[43] Dürre P. (2014). Physiology and Sporulation in Clostridium. Microbiol. Spectr..

[44] Guo P., Zhang K., Ma X., He P. (2020). *Clostridium* species as probiotics: Potentials and challenges. J. Anim. Sci. Biotechnol..

[45] Jessen G.L., Lichtschlag A., Struck U., Boetius A. (2016). Distribution and composition of thiotrophic mats in the hypoxic zone of the black sea (150–170 m Water Depth, Crimea Margin). Front. Microbiol..

[46] Sharndama H.C., Mba I.E. (2022). Helicobacter pylori: An up-to-date overview on the virulence and pathogenesis mechanisms. Braz. J. Microbiol..

[47] Pirzadeh M., Khalili N., Rezaei N. (2022). The interplay between aryl hydrocarbon receptor, H. pylori, tryptophan, and arginine in the pathogenesis of gastric cancer. Int. Rev. Immunol..

[48] Waskito L.A., Salama N.R., Yamaoka Y. (2018). Pathogenesis of helicobacter pylori infection. Helicobacter.

[49] Wu Y., Peng X., Li X., Li D., Tan Z., Yu R. (2022). Sex hormones influence the intestinal microbiota composition in mice. Front. Microbiol..

[50] Zhou K., Deng N., Yi X., Cai Y., Peng M., Xiao N. (2022). Baohe pill decoction for diarrhea induced by high-fat and high-protein diet is associated with the structure of lactase-producing bacterial community. Front. Cell. Infect. Microbiol..

[51] Fang L., Shen J., Wu Y., Tan Z. (2025). Involvement of intestinal mucosal microbiota in adenine-induced liver function injury. 3 Biotech.

[52] Shen J., Fang L., Tan Z., Xiao N., Peng M. (2025). The Effects of Functional Biscuits on Intestinal Mucosal Microbiota Composition, Brain Function, and Antioxidant Activity. Biosci. Microbiota Food Health.

[53] Liu J., Qiao B., Cai Y., Tan Z., Deng N. (2023). Diarrhea accompanies intestinal inflammation and intestinal mucosal microbiota dysbiosis during fatigue combined with a high-fat diet. BMC Microbiol..

[54] Zhou M., Li X., Wang X., Deng N., Cai Y., Tan Z. (2024). The dysfunction in intestinal microorganisms and enzyme activity as significant contributors to diarrhea with kidney-yang deficiency syndrome. Front. Microbiol..

[55] Schnürer J., Rosswall T. (1982). Fluorescein diacetate hydrolysis as a measure of total microbial activity in soil and litter. Appl. Environ. Microbiol..

[56] Hui H., Wu Y., Zheng T., Zhou S., Tan Z. (2020). Bacterial Characteristics in Intestinal Contents of Antibiotic-Associated Diarrhea Mice Treated with Qiweibaizhu Powder. Med. Sci. Monit..

